# Impact of Endodontic Treatment on CRP Levels in Apical Periodontitis: A Prospective Observational Cohort Study

**DOI:** 10.3390/jcm14248929

**Published:** 2025-12-17

**Authors:** Hussain Al akam, Asaad Abdulrahman Abduljawad, Basel Abozor

**Affiliations:** 1 Private Practice, 159 E North Ave, Northlake, IL 60164, USA; alakamhussain@gmail.com (H.A.a.);; 2 Public Health Department, College of Health Sciences, Umm Al-Qura University, Makkah 11343, Saudi Arabia

**Keywords:** C-reactive protein, apical periodontitis, endodontic retreatment, systemic inflammation, oral–systemic health

## Abstract

**Background/Objectives:** Apical periodontitis is a prevalent dental condition associated with systemic inflammation. C-reactive protein (CRP) is a sensitive biomarker of inflammatory status. While previous studies have examined CRP changes after endodontic treatment, limited evidence exists on short-term systemic effects following clinically indicated endodontic therapy in healthy individuals. This prospective observational cohort study aimed to evaluate the impact of endodontic treatment or retreatment on serum CRP levels in patients with symptomatic and asymptomatic apical periodontitis and assess demographic influences. No control group was included. **Methods:** A prospective observational cohort study was conducted in a private endodontic clinic in Jeddah, Saudi Arabia (2021–2023). Three hundred ASA I patients were enrolled and categorized into symptomatic apical periodontitis (SAP) and asymptomatic apical periodontitis (AAP) groups. Blood samples were collected before treatment and two weeks post-treatment. CRP levels were measured using a high-sensitivity assay. Statistical analyses included paired *t*-tests, Mann–Whitney U tests, and multiple regression. Laboratory personnel were blinded to patient grouping. **Results:** CRP levels significantly decreased after treatment in both groups (SAP: 6.99 to 2.01 mg/L; AAP: 5.40 to 1.64 mg/L; *p* < 0.001). Reduction was greater in SAP (mean difference = 4.98 mg/L) than AAP (3.76 mg/L; *p* < 0.001). Paired *t*-test showed a very large effect size (Cohen’s d = 3.51). Age and sex were not significant predictors of CRP changes (R^2^ < 0.02). **Conclusions:** Endodontic treatment or retreatment significantly reduces systemic inflammation in patients with apical periodontitis. These findings reinforce the oral–systemic health link and highlight the clinical relevance of managing apical infections. Longer-term studies and inclusion of additional biomarkers are recommended.

## 1. Introduction

Apical periodontitis is a localized inflammation of the peri-radicular tissues due to the existence of bacteria and bacterial toxins from contaminated root canals [1,2]. It can remain asymptomatic for extended periods and is often discovered incidentally during routine radiographic examinations [3,4]. Tibúrcio-Machado et al. described the presence of apical periodontitis in over than 50% of adults globally, and that prevalence is associated with the presence of systemic diseases [4].

C-reactive protein (CRP) is a sensitive biomarker of systemic inflammation and is widely used to assess inflammatory burden. Elevated CRP levels have been linked to oral infections, including periodontal and endodontic diseases, and may reflect the systemic impact of localized dental pathology.

Several studies have explored this relationship. For example, Valiyaveetil Karattil et al. [5] discovered that CRP levels in the serum are related to periodontal disease, and periodontitis severity directly affects the former. These findings position CRP as a sensitive marker for the presence and severity of oral infections. Another study [6] compared CRP levels in serum of patients with odontogenic infections before and 7 days after elimination of the infection source. The author revealed that the presence of an odontogenic infection was related to increased serum CRP levels, which decreased 7 days after tooth extraction [6]. Similar results were reported in another study, confirming that elevated CRP levels are infection severity- and type-dependent [7].

A retrospective study in endodontics examined levels of CRP and other inflammatory markers in teeth affected by pulpitis. Researchers measured these before and one week after root canal treatment. It was found that CRP and other markers decreased after that week. The study did not account for the periapical condition or the initial diagnosis before treatment. This highlights the need for more comprehensive assessments in future research [8].

Previous studies have investigated the correlation between systemic diseases and the occurrence of apical periodontitis. Evidence indicates a clear link between apical periodontitis and various systemic conditions. These include diabetes [9], rheumatoid arthritis [10], osteoporosis [11], inflammatory bowel diseases [12], and atherosclerosis [13]. Al-Zahrani et al. [14] examined the link between apical periodontitis and serum CRP levels in diabetic patients. They found that apical periodontitis correlates with poorly controlled diabetes and elevated CRP levels.

Another study reported a significant reduction in periapical index (PAI) scores after treatment. It also indicated improved periapical health [15].

Bakhsh et al. also examined changes in various inflammatory markers, such as CRP levels, among patients with apical periodontitis who received endodontic retreatment or apicoectomy. The researchers noted elevated CRP levels during the initial three months. These levels then declined back to baseline by the one-year follow-up [16].

Another long-term study observed reduced CRP levels. This study included follow-up on CRP levels in patients who had nonsurgical endodontic retreatment or periapical surgery. The follow-up occurred 2 years after treatment [17].

However, the specific impact of root canal retreatment on levels of CRP in patients with apical periodontitis has not been fully explored.

Demographic factors, such as age and sex, might play a key role in explaining variations in C-reactive protein (CRP) levels and how they respond to endodontic treatment. Women exhibit higher CRP levels than men. These levels also tend to increase with age. This pattern points to a stronger inflammatory response in these groups. This underscores the notion that racial and ethnic factors influence CRP levels. Non-white groups show higher levels. Socioeconomic status plays a role too. Lower socioeconomic groups exhibit elevated CRP levels [18]. Understanding these differences is essential for designing targeted interventions to improve patient outcomes.

Although previous studies have examined CRP changes after primary endodontic treatment, evidence on short-term systemic effects following endodontic retreatment remains scarce. Most available studies involve small samples or long-term follow-up, leaving a gap in understanding immediate systemic responses to clinically indicated retreatment in healthy individuals.

This study aimed to evaluate the short-term impact of endodontic treatment or retreatment on serum CRP levels in patients with symptomatic and asymptomatic apical periodontitis and to assess demographic influences.

## 2. Methods

This study followed the STROBE guidelines (Strengthening the Reporting of Observational Studies in Epidemiology) to ensure clear and transparent reporting, maintain high standards, and improve the overall quality of reporting. The manuscript underwent AI-assisted proofreading using Grammarly for improved clarity and accuracy.

### 2.1. Sampling and Patient Selection

This prospective observational cohort study took place in an endodontic private clinic in Jeddah, Saudi Arabia, from August 2021 to August 2023.

Sample size was calculated using G*Power (version 3.1.9.7) for a paired *t*-test with an assumed effect size (dz) of 0.5, α = 0.05, and power = 0.80, indicating that 27 participants would be sufficient to detect significant changes in CRP levels. However, we enrolled 300 patients to improve precision, allow subgroup analysis (SAP vs. AAP), and enhance external validity. Multiple comparisons were limited to predefined hypotheses; therefore, adjustments were not required.

During the study period, 1469 patients received treatment. The first 300 who met the inclusion criteria were selected after signing the necessary consent forms. The patient sample consisted of 143 females and 157 males.

Data was extracted from patient records on variables such as demographic characteristics, CRP levels, and periapical index scores. CRP levels were measured using a high-sensitivity CRP assay.

Quality control measures included duplicate testing of blood samples, regular calibration of laboratory equipment, and adherence to standardized sample collection and analysis protocols.

There was no missing data for any of the variables. All participants provided complete data, including CRP levels and demographic information, ensuring the integrity of the statistical analyses. Therefore, no other methods for handling missing data were necessary. All patients returned for follow-up because they were scheduled for pre-paid prosthetic visits, which minimized dropout and ensured complete data collection.

This study was approved by the Institutional Review Board of the private clinics, Jeddah, Saudi Arabia (approval date: 14 January 2021). All participants provided informed consent in English and Arabic before enrollment.

Statistical analyses were performed using IBM SPSS 25 to examine the association between CRP levels and apical periodontitis and the effect of demographic factors on CRP levels. The Shapiro–Wilk test with *p*-values of *p* < 0.05 was used to determine if the data were normally distributed. Student’s *t*-test was used to compare two related groups or paired data.

In addition to parametric tests, non-parametric analyses were performed to confirm robustness. The Wilcoxon signed-rank test was applied for paired comparisons of CRP levels before and after treatment. The Mann–Whitney U test was used to compare CRP levels and CRP reduction between symptomatic apical periodontitis (SAP) and asymptomatic apical periodontitis (AAP) groups.

Sensitivity analyses were conducted to assess the robustness of the study findings by varying the inclusion criteria and reanalyzing the data. The results remained consistent, indicating that the results are reliable and were not influenced by specific assumptions or criteria. The inclusion and exclusion criteria is shown in Table 1.

The presence of apical periodontitis (SAP or AAP) was diagnosed per AAE guidelines. SAP was identified by pain on percussion or palpation with radiographic evidence of apical radiolucency, while AAP was diagnosed based on radiolucency without pain. SAP and AAP represent periapical diagnoses, whereas irreversible pulpitis, pulp necrosis, and previously treated teeth represent pulpal diagnoses included as underlying pathology leading to apical involvement. The study focused on periapical status (SAP vs. AAP) regardless of whether the case involved primary treatment or retreatment, as the primary objective was to assess systemic inflammatory changes based on periapical condition.

Patients using medications that influence bone metabolism, e.g., bisphosphonates, immunosuppressants, selective serotonin reuptake inhibitors, and hormone replacement therapy, were excluded [23].

Obese patients were excluded because obesity is associated with elevated CRP levels, which could confound interpretation of systemic inflammatory changes related to apical periodontitis. Similarly, patients with hospitalization within the past 24 months were excluded because hospitalization is often associated with higher ASA classifications, which would conflict with the inclusion criterion of ASA I status [24].

### 2.2. Blood Collection

After determining the patients’ eligibility to participate in the study and obtaining the proper consent, blood samples were collected using a gold top tube. The samples were then labeled using special coding that referred to each patient without exposing any personal identifying data. The samples were then transported to the laboratory and processed for CRP analysis.

All analyses were performed in the same clinic complex laboratory using a high-sensitivity CRP assay on the EUROLyser Smart 546 analyzer (Eurolyser Diagnostica GmbH, Salzburg, Austria).

Blood samples were collected before endodontic re/treatment and again two weeks later during the scheduled buildup/crown preparation visit. This timing minimized patient dropout and allowed assessment of early systemic inflammatory changes following therapy.

### 2.3. Blinding Considerations

Clinician blinding was not feasible in this study because the operator was responsible for performing endodontic treatment or retreatment, which required procedural decisions based on the tooth’s condition. To minimize potential bias, all treatments were standardized and performed by the same experienced clinician following a strict protocol. Additionally, outcome assessment was blinded: laboratory personnel analyzing CRP samples were unaware of patient grouping, and statistical analyses were conducted independently. These measures reduce detection and analytical bias despite the absence of operator blinding.

### 2.4. Root Canal Treatment or Retreatment

Treatment or retreatment was performed by the same practitioner as follows:

A local anesthetic was administered and rubber dam isolation was applied. Local anesthesia was achieved using 2% lidocaine with 1:100,000 epinephrine (Septodont, Lancaster, PA, USA) for all patients.

After excavating the caries and pulp chamber deroofing, access was gained to the canals, and working lengths were determined using an apex locator. Radiographs were acquired to confirm working lengths, which was performed for all patients to ensure accuracy and prevent procedural errors, as recommended by the American Association of Endodontists (AAE) and supported by clinical guidelines [25,26].

The ProTaper Gold (Maillefer Instruments Holding SARL, Ballaigues, Switzerland) rotary system was used to clean and shape the canals, with 6% NaOCl and RC prep used throughout the procedure. Brasseler Endosequence BC (Gebr. Brasseler GmbH & Co. KG, Lemgo, Germany) sealer was applied to the gutta-percha cone and seated. Excess sealer and gutta-percha were removed to the level of the canal orifice. Final radiographs were obtained. For nonsurgical retreatment, the gutta-percha was removed, the canal(s) were disinfected with 2% Chlorhexidine, and the previous procedure for obturation was performed as described above. The use of 2% chlorhexidine as an irrigant in retreatment cases is supported by its broad-spectrum antimicrobial activity and substantivity, making it effective against resistant microorganisms such as Enterococcus faecalis [27,28].

Furthermore, Zandi et al. (2019) reported that chlorhexidine has similar disinfecting properties to sodium hypochlorite, with the advantage of fewer potential adverse effects [29].

Patients were scheduled for 1 h appointments, and procedures did not exceed this duration. All treatments were performed by one experienced endodontist throughout the 2-year study period. The irrigation protocol included 6% NaOCl between each hand and rotary file, with final activation using an ultrasonic tip. For retreatment cases, after gutta-percha removal, 2% Chlorhexidine irrigation was performed, followed by saline irrigation, then 6% NaOCl with ultrasonic activation. The second visit after two weeks was scheduled for final buildup and crown preparation (if applicable) with the general dentist.

The teeth were etched with 37% phosphoric acid for 15 s, rinsed, and air-dried. I-Bond was applied, air-thinned, and cured for 20 s. Flowable composite was used as an orifice barrier, and the teeth were temporarily restored with a glass ionomer. Patients were brought back for buildup appointments two weeks after the root canal re/treatment appointment.

## 3. Results

### 3.1. Baseline Characteristics

All 300 patients (143 females, 157 males) completed the study and provided full data. Baseline age and PAI scores did not differ significantly between symptomatic apical periodontitis (SAP) and asymptomatic apical periodontitis (AAP) groups (*p* > 0.40), indicating comparable initial characteristics.

### 3.2. Reduction in CRP Levels After Endodontic Treatment

As shown in Table 2, endodontic treatment produced a substantial and statistically significant decrease in CRP levels across the full sample (mean reduction: 4.66 mg/L; 95% CI: 4.51–4.82; *p* < 0.001; Cohen’s d = 3.51).

SAP: CRP decreased from 6.99 mg/L to 2.01 mg/L (mean reduction: 4.98 mg/L).

AAP: CRP decreased from 5.40 mg/L to 1.64 mg/L (mean reduction: 3.76 mg/L).

These changes correspond to a 65–70% reduction in systemic inflammatory burden, demonstrating a rapid systemic response following removal of the apical infection source.

### 3.3. Comparison Between SAP and AAP

**Figure 1:**
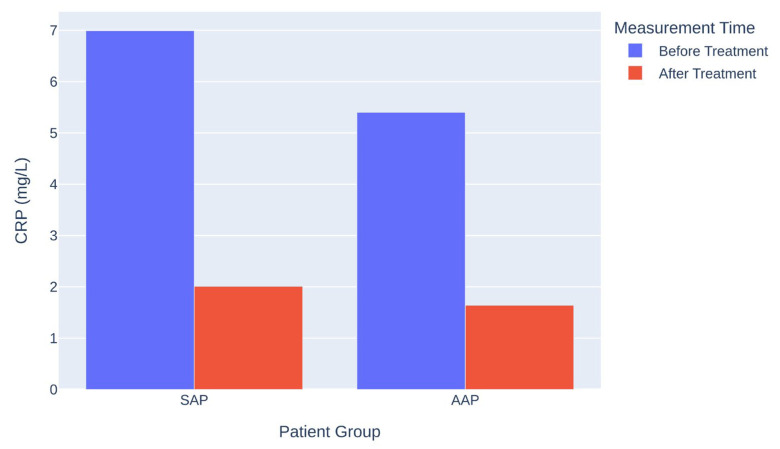
CRP levels before and after endodontic treatment.

Before treatment, CRP levels were significantly higher in SAP than AAP (*p* < 0.001). After treatment, both groups showed marked improvement, but SAP demonstrated a significantly greater reduction (mean difference = 1.22 mg/L; 95% CI: 0.90–1.53; *p* < 0.001; Cohen’s d = 1.00), illustrated in Figure 1.

### 3.4. Influence of Age and Sex

As shown in Table 3, multiple regression analysis showed that age and sex were not significant predictors of baseline CRP, post-treatment CRP, or the magnitude of CRP reduction (*p* > 0.05; R^2^ < 0.02), indicating minimal influence of demographic factors in this ASA I cohort.

### 3.5. Sensitivity Analyses

Non-parametric tests (Wilcoxon signed-rank and Mann–Whitney U) confirmed all primary findings, supporting the robustness of the results.

The Periapical Index (PAI) assessment: PAI scores were assessed to evaluate radiographic healing and compare periapical status between SAP and AAP groups. This comparison provides insight into local disease resolution following endodontic treatment and complements systemic CRP findings, as shown in Table 4.

#### Clinical Relevance

These findings underscore the importance of timely endodontic intervention to reduce systemic inflammation and potentially mitigate associated systemic health risks.

## 4. Discussions

A 65–70% reduction in CRP after endodontic treatment reinforces the oral–systemic health link and underscores the importance of timely intervention. The study focused on comparing symptomatic apical periodontitis (SAP) and asymptomatic apical periodontitis (AAP) groups, regardless of whether the case involved primary treatment or retreatment. Therefore, separate data for primary versus retreatment cases were not analyzed, as the primary objective was to assess systemic inflammatory changes based on periapical status rather than pulpal condition. This design choice ensured that the analysis remained focused on periapical status, but it may limit comparisons between primary and retreatment cases.

Furthermore, this study provides strong evidence (Cohen’s d = 3.51) that endodontic treatment significantly reduces systemic inflammation markers, reinforcing the oral–systemic health link. The inclusion of 95% confidence intervals for mean CRP values and differences enhances the precision and interpretability of our results. These intervals provide a range within which the true effect likely lies, reinforcing the robustness of the observed reductions and supporting clinical relevance.

Similar systemic effects of odontogenic infections have been reported in cases of cervico-facial phlegmons, where treatment significantly modulated systemic immune-inflammatory indices [30]. These findings further support the systemic impact of oral infections and complement our observations on CRP reduction following endodontic therapy.

The connection among age, sex, and CRP levels indicates that these demographic elements are unlikely to influence variations in CRP levels. Therefore, further studies are essential to clarify the underlying processes and to pinpoint possible confounding variables.

A previous study conducted by Hughes and Kumari identified a connection between age and elevated levels of CRP. They proposed that this association likely stems from older individuals being more prone to underlying inflammation or chronic health conditions, which in turn contribute to higher CRP concentrations [31]. Given that this study included only healthy patients classified as ASA I, the absence of a significant association between age and CRP levels before and after endodontic re-treatment can be readily explained. These participants were generally free of systemic conditions, and age likely did not exert a noticeable influence on inflammation markers such as CRP. Gender differences were observed in CRP level changes following treatment. The estimate, however, carried a high standard error compared to the effect size. This points to a degree of uncertainty in the results. These observations contrast with prior research, which indicated markedly higher CRP levels among females than males. Overall, the findings highlight the need for further investigation into these variations [32]. This underscores the need for further research to investigate potential confounding factors. They would also clarify how demographic variables interact with systemic inflammation during endodontic treatment.

This study’s findings match the results from various follow-up studies. Those studies have looked at CRP levels after non-surgical root canal retreatment [16]. This study arrived at a similar final outcome as the one reported by Bakhsh et al. It demonstrated a faster reduction in CRP levels. In contrast, Bakhsh et al. observed a temporary rise in CRP levels before they decreased [16]. This difference needs further investigation to understand the complicated healing process mechanism following endodontic retreatment.

In contrast, the results of this study align with those of previous research that examined the link between the presence of oral or dental infections and increased CRP levels [5,6,7,8]. Previous studies have examined the relationship between SAP and inflammatory responses in the body. However, further research is needed to explain the significant CRP level reductions compared to those in AAP.

Some limitations of this study included its short follow-up study design, which prevented the establishment of a causal relationship between apical periodontitis and CRP levels, as well as the lack of operator blinding, single-population focus, and assessment of only CRP. Future studies should include longer follow-up, multiple cities, and more diverse populations with additional biomarkers.

For that, suggested longer longitudinal studies are needed to confirm temporal relationships and causality. CRP was measured for this duration, since longer follow-up periods were not possible due to patient scheduling constraints. Therefore, findings reflect short-term inflammatory changes rather than long-term systemic effects.

Another limitation is the inability to blind the clinician performing the treatment. Operator blinding was not feasible because the experienced endodontics practitioner needed to make procedural decisions during endodontic treatment or retreatment. To minimize bias, all treatments were standardized and performed by the same experienced clinician following a strict protocol, and outcome assessment was blinded at the laboratory level. These measures reduce detection and analytical bias despite the absence of operator blinding.

Furthermore, the absence of a healthy control group to avoid unnecessary interventions in healthy individuals limits causal inference.

Another added limitation is that this study focused on Saudi patients, which may limit the generalization of the findings to other populations. Future studies with more diverse populations should be conducted to enhance the external result validity. Furthermore, this study did not consider any other inflammatory markers that could provide a more comprehensive understanding of the systemic inflammatory response to endodontic treatment. Future studies with a broader range of biomarkers are needed to explain the underlying mechanisms. Finally, certain factors not accounted for in this study, such as diet, lifestyle habits, genetic predisposition, and other underlying health conditions, may have influenced CRP levels and potentially affected the results. These unaddressed variables should be considered in future research to strengthen the findings [33].

Future studies that account for these variables will provide a clearer understanding of how endodontic retreatment impacts systemic inflammation. By controlling for these confounding factors, researchers can obtain more accurate, reliable, and meaningful results.

## 5. Conclusions

Endodontic treatment or retreatment significantly reduces systemic inflammation in patients with apical periodontitis, supporting the oral–systemic health link. Further studies with longer follow-up and additional biomarkers are recommended to confirm these findings and explore underlying mechanisms.

## Figures and Tables

**Table 1:** Inclusion and exclusion criteria.

Category	Criteria
The Inclusion Criteria	ASA I patients: ASA I refers to patients classified by the American Society of Anesthesiologists Physical Status Classification System as healthy individuals with no systemic disease, non-smokers, minimal alcohol use, and normal BMI [19].
	Presence of apical periodontitis (symptomatic or asymptomatic) diagnosed per AAE guidelines
	One tooth requiring endodontic treatment or retreatment with pulpal diagnoses: symptomatic/asymptomatic irreversible pulpitis, pulp necrosis, or previously treated. The inclusion of these pulpal diagnoses was necessary because the primary focus of this study was on the condition of the periapical tissues (symptomatic or asymptomatic apical periodontitis), not the pulpal status. This approach ensured that all cases had apical involvement regardless of pulp condition
	Clearance of periodontal and gingival inflammation by a general dentist: This criterion was included to eliminate the potential confounding effects of periodontal disease on CRP levels, as periodontal inflammation is known to significantly influence systemic inflammatory markers [20].
The Exclusion Criteria	Patients with systemic disease
	Normal periapical region (radiographically intact periapical tissues), referring to radiographically intact periapical tissues without any signs of radiolucency, as assessed according to AAE guidelines.
	Recent hospitalization within the past 24 months
	Moderate or severe marginal periodontal disease
	Antibiotic or corticosteroid use within the previous 3 months: The three-month exclusion period was chosen to minimize residual systemic effects of antibiotics and corticosteroids on inflammatory markers such as CRP. Evidence indicates that CRP levels typically normalize within 6–8 weeks after cessation of anti-inflammatory or antimicrobial therapy, and some studies recommend a conservative window of up to three months to ensure complete washout [21,22].
	Medications affecting bone metabolism (e.g., bisphosphonates, SSRIs, HRT)
	Smokers
	Pregnant women
	Obesity
	Refusal to provide blood samples
	Inability to attend follow-up appointments

**Table 2:** Summary of CRP changes.

Group	CRP Before (mg/L)	CRP After (mg/L)	Mean Difference (mg/L)	*p*-Value
SAP	6.99	2.01	4.98	<0.001
AAP	5.40	1.64	3.76	<0.001

**Table 3:** Demographic influence.

Variable	*p*-Value	Interpretation
Age	>0.05	Not a significant predictor
Sex	>0.05	Not a significant predictor

**Table 4:** Periapical Index (PAI) comparison between SAP and AAP groups.

Group	PAI Score (Mean ± SD)	N	Std Error Mean
AAP	2.67 ± 1.040	78	0.118
SAP	2.78 ± 1.033	222	0.068

## Data Availability

The data supporting the findings of this study are available from the corresponding author upon reasonable request.
